# Genome-Wide Association Study Identifies Novel Susceptibility Genes Associated with Coronary Artery Aneurysm Formation in Kawasaki Disease

**DOI:** 10.1371/journal.pone.0154943

**Published:** 2016-05-12

**Authors:** Ho-Chang Kuo, Sung-Chou Li, Mindy Ming-Huey Guo, Ying-Hsien Huang, Hong-Ren Yu, Fu-Chen Huang, Fuyong Jiao, Hsing-Chun Kuo, Jorge Andrade, Wen-Ching Chan

**Affiliations:** 1 Department of Pediatrics and Kawasaki Disease Center, Kaohsiung Chang Gung Memorial Hospital and Chang Gung University College of Medicine, Kaohsiung, Taiwan; 2 Genomics and Proteomics Core Laboratory, Department of Medical Research, Kaohsiung Chang Gung Memorial Hospital and Chang Gung University College of Medicine, Kaohsiung, Taiwan; 3 Children's Hospital of Shaanxi Provincial People's Hospital and Jiaotong University, Xi'an, China; 4 Department of Nursing, Chang Gung University of Science and Technology, Chiayi, Taiwan; 5 Center for Research Informatics, The University of Chicago, Chicago, Illinois, 60637, United States of America; Weill Medical College of Cornell University, UNITED STATES

## Abstract

Kawasaki disease (KD) or Kawasaki syndrome is known as a vasculitis of small to medium-sized vessels, and coronary arteries are predominantly involved in childhood. Generally, 20–25% of untreated with IVIG and 3–5% of treated KD patients have been developed coronary artery lesions (CALs), such as dilatation and aneurysm. Understanding how coronary artery aneurysms (CAAs) are established and maintained in KD patients is therefore of great importance. Upon our previous genotyping data of 157 valid KD subjects, a genome-wide association study (GWAS) has been conducted among 11 (7%) CAA-developed KD patients to reveal five significant genetic variants passed pre-defined thresholds and resulted in two novel susceptibility protein-coding genes, which are NEBL (rs16921209 (P = 7.44 × 10^−9^; OR = 32.22) and rs7922552 (P = 8.43 × 10^−9^; OR = 32.0)) and TUBA3C (rs17076896 (P = 8.04 × 10^−9^; OR = 21.03)). Their known functions have been reported to associate with cardiac muscle and tubulin, respectively. As a result, this might imply their putative roles of establishing CAAs during KD progression. Additionally, various model analyses have been utilized to determine dominant and recessive inheritance patterns of identified susceptibility mutations. Finally, all susceptibility genes hit by significant genetic variants were further investigated and the top three representative gene-ontology (GO) clusters were regulation of cell projection organization, neuron recognition, and peptidyl-threonine phosphorylation. Our results help to depict the potential routes of the pathogenesis of CAAs in KD patients and will facilitate researchers to improve the diagnosis and prognosis of KD in personalized medicine.

## Introduction

Kawasaki disease (KD; OMIM 611775), also known as mucocutaneous lymph node syndrome, or Kawasaki syndrome, is one of the most common systemic vasculitis illnesses that preferentially occur in children younger than 5 years old. KD was first described by Dr. Tomisaku Kawasaki in 1967, and its diagnosis in clinics is primarily based upon a history of prolonged fever for at least five days along with four or more of the following manifestations: oral mucosa changes, conjunctivitis, enlargement of the cervical lymph nodes, swelling of the hands and feet and polymorphous skin rashes [1]. Up to date, higher incidence rates of KD have been reported in Asian populations; such as Japanese, Koreans, Taiwanese, and China, up to 239.6, 113.1, 66.2, and 49.4 per 100,000 children under five years old, respectively. Although the ethnic dominant pattern in KD has been identified, the incidence rate and the total number of patients with KD have been continuously increasing all over the place. Taken Together, this ethnic-preferential pattern implies that genetic factors might play a critical role as well as the environmental influence in the development and maintenance of KD in those susceptible hosts on genome scale.

As a vasculitis of the small to medium vessels, KD has a predilection for the involvement of the coronary arteries. Up to 25% of patients with KD may develop coronary artery lesions (CALs) if not given adequate treatment with intravenous immunoglobulin (IVIG), which in turn greatly increases the risk of coronary artery aneurysms (CAAs), and subsequent coronary artery thrombosis or myocardial infarction [2]. However, according to the current American Heart Association (AHA) guidelines, treatment with high-dose IVIG during the acute phase of the self-limited vasculitis in KD can substantially reduce the risk of coronary artery formation to 3–5% [3]. Namely, delayed diagnosis of KD and late treatment with IVIG is one of the critical risk factors for the development of CALs [4]. As an important complication of KD, other risk factors associated with CALs development include: children that are younger than 1 years old, prolonged fever duration or those who require more than one dose of IVIG, and those with higher inflammatory markers at baseline [5–8].

Although the underlying etiology of KD remains largely uncharacterized, clinical and epidemiology evidence indicates that an inflammatory response has been induced due to a ubiquitous infectious factor, subsequently host immune dysregulation frequently occurred in a small subset of genetically predisposed children. In immunopathogenosis, the activation of innate and acquired immunity has been reported to associate with KD patients in human and animal studies. In children with KD, CAAs usually develop within the first 4–6 weeks after disease onset [9]. Specifically, increased neutrophilic infiltration of the coronary vessels walls occur initially in the first two weeks after KD onset [10], followed by higher infiltration rate of natural killer cells and CD8 T cells afterwards [11]. On the other hand, from gene perspective, tumor necrosis factor α (TNF-α) appears to be a crucial mediator of inflammatory response in KD patients, by up-regulating the transcription of matrix metalloproteinases (MMP) such as MMP-9, which in turn leads to increased vessel wall elastin degradation and CAA formation [12]. Therapeutic blockage of TNF-α in murine model prevents the development of coronary artery disease, and has been used as an alternative therapy for children with IVIG-resistance in KD [12, 13].

The power of genome-wide association study (GWAS) makes it as one of the commonly used approaches to detect genomic loci individually or coincidently associated with the disease of interest in one high-throughput experiment on a genome-wide scale. In recent years, numerous GWASs have been conducted to identify single nucleotide polymorphisms (SNPs) significantly associated with the occurrence of KD among different populations, further resulting in the discovery of a number of susceptibility genes and their potential roles in the development and maintenance of KD [14–19]. It gives rise to a handful of SNPs being consistently reported in different GWASs of KD across different ethnics. For instance, *rs28493229* and *rs2290692* in ITPKC [15, 20–23], *rs113420705* (formerly *rs72689236*) in CASP3 [24–27], *rs1801274* in FCGR2A [15, 19, 28–31], *rs4813003* and *rs1569723* in CD40 [18, 19, 31], and *rs2736340*, *rs2254546* and *rs2618476* in BLK [18, 19, 28, 30–32] have been frequently revealed as significant genetic variants associated with the occurrence of KD in various GWASs. DNA methylation array data also identified an association between genomic hypomethylation of FCGR2A and susceptibility to KD and IVIG resistance [33]. Additionally, when considering the different outcomes upon KD subjects; for instance, the presence of CALs [16, 25, 34–39]/CAAs [40–45] or IVIG resistance [25, 26, 34, 46], a number of studies have been developed aiming to uncover the association between genetic variants and KD. Most of these studies inspected individual susceptibility genes and corresponding SNPs involved in the regulatory network of immune responses as well as cardiovascular-associated pathogenesis that might contribute to the formation of different outcomes or the response of treatments in KD.

The objectives of this study are to determine the genetic differences in CAA-developed (CAA+) KD patients using non-CAA developed (CAA-) KD as control, and further imply underlying molecular mechanisms, by which susceptibility genes located by significant polymorphisms might be associated with cardiac dysfunction. In this study, a comprehensive GWAS has been conducted guided by our previous high throughput genotyping microarray data [18] to reveal susceptibility loci associated with the development and maintenance of CAA in KD. The result of this study may be of interest to researchers in the KD community attempting to develop a more precise diagnosis or even prognosis of CAAs in KD patients.

## Methods

### Ethical statement

This study was approved by the Ethics Committee of the Institutional Review Board in Kaohsiung Chang Gung Memorial Hospital in Taiwan. Written informed consents were acquired from the Kawasaki disease patients’ parents or guardians according to institutional requirements and Declaration of Helsinki principles.

### Study subjects and phenotype definition

Individuals fulfilling the diagnostic criteria of KD (n = 183) (including 146 patients without CAAs, 11 patients with CAAs, and 26 unclassified ones in our previous study [18]) were consecutively identified and recruited from Kaohsiung Chang Gung Memorial Hospital, Taiwan. CAA complications were determined from the echocardiograms according to the criteria provided by the Japanese Kawasaki Disease Research Committee: coronary arteries were identified as abnormal if the internal lumen diameter was ≥3 mm in children <5 years old or ≥4 mm in children ≥5 years old, if the internal diameter of a segment measured ≥1.5 times that of an adjacent segment, or if the coronary lumen was apparently irregular and the lesions were till noted 8 weeks after disease onset. Transient dilation of coronary artery was excluded.

### SNP genotyping and quality control

Genomic DNA was extracted from blood using the Puregene DNA Isolation Kit (Gentra Systems). Each individual was genotyped using the Affymetrix Genome-Wide Human SNP Array 6.0 (with a total of 906,600 SNPs) according to the manufacturer’s protocols by the National Center for Genome Medicine (NCGM) at Academia Sinica, Taiwan [18]. Three major criteria were utilized to qualify valid SNPs including call rate (CR) >99%, minor allele frequency (MAF) >0.01, and the p-value of Hardy-Weinberg equilibrium (HWE) >1 × 10^−4^.

### Statistical analysis

The allelic and genotypic frequency distributions of polymorphisms in KD patients according to the presence or absence of CAAs were determined through chi-square and Fisher’s exact analysis using PLINK software (v1.07, 10/Aug/2009) [47], respectively. Genotypes were obtained from direct counting followed by allele frequency calculations variables, and odds ratios (ORs) were calculated from allelic frequencies with a 95% confidence interval (95% CI). P-value of less than 0.001 was considered statistically significant, and adherence to the HWE constant was evaluated using a chi-square test with one degree of freedom. Furthermore, the dominant, recessive, and general genotypic models were employed to determine the genetic inheritance patterns of significant SNP candidates to susceptibility of CAA formation in KD patients [48]. Gene-ontology (GO) enrichment analysis has been calculated using chi-square distribution, and the collection of GO terms were further summarized into GO clusters using the web-based tool of REVIGO [49].

## Results

### Genetic association analysis between individual SNP and the risk of CAA complications in KD patients

**Fig 1:**
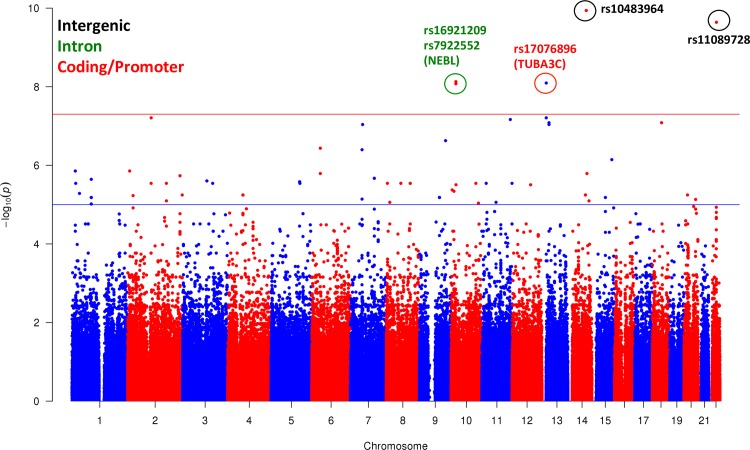
Manhattan plot of the association between SNPs and susceptibility of CAA formation in KD. The −log_10_ *P*-values of significance shown in chromosomal order for qualified 559,602 SNPs tested for association in initial sample of 11 KD patients with CAA and 146 KD ones without CAA. The x-axis represents each of the SNPs used in the primary scan according to their genomic location. The y-axis indicates the −log_10_ *P*-values of the trend test. Horizontal lines indicate the general and stringent thresholds of −log_10_ *P* as 5 and 7, respectively.

To identify susceptibility genes associated with KD-associated CAAs, the presence and absence of CAAs occurred in 183 Taiwanese KD patients were linked toward our previous genotyping data [18]. After excluding 26 (14.21%) KD subjects without the determination of developing CAAs, 157 (85.79%) KD cases with the determination of CAA formation as listed in Table 1 were used in further analysis. In this study, a comprehensive GWAS has been applied to these valid genotyping data of KD patients consist of 96 (61.15%) males and 61 (38.85%) females (Table 1). In total, eleven (7.01%) KD patients with CAA formation were compared to the rest of 146 (92.99%) KD patients without CAA formation to identify significant genetic polymorphisms associated with the development and maintenance of CAAs in KD. Before identifying significant SNPs, customized criteria; including minor allele frequency (MAF), call rate (CR), and p-values of Hardy-Weinberg equilibrium (HWE), were applied to filter uncertain SNPs as described in the Methods, resulting in 559,609 (64.46%) out of 868,153 genotyped variants in the array remained and further qualified for the follow-up analysis. The genome-wide association results were plotted throughout chromosomes according to p-values calculated using chi-square tests as significance (Fig 1).

**Table 1:** Subject statistics used in this study.

Phenotype/Sex	CAA+	CAA-	Sum
**Female**	5 (3.18)	56 (35.67)	61 (38.85)
**Male**	6 (3.82)	90 (57.32)	96 (61.15)
**Sum**	11 (7.01)	146 (92.99)	157

KD patients were divided into two groups according to the presence or absence of CAAs as CAA+ and CAA-, respectively.

A p-value of less than 0.001 was used as the threshold to specify the final set of significant genetic variants associated with CAA formation in KD patients, which in turn 720 (0.13%) sites were qualified for further analyses. When mapping these significant genetic polymorphisms toward gene structures, 31 (4.31%), 289 (40.14%), and 400 (55.56%) SNPs were identified to locate in genic, intronic, and intergenic regions, respectively. In addition, to identify potential susceptibility genes of the development of CAAs in KD patients, the gene hits of the top ranking non-intergenic SNPs were further investigated. The unique collection of genes located by significant genetic variants includes NEBL (Nebulette sarcomeric isoform; chr10:21,110,094–21,226,537), TUBA3C (Tubulin, Alpha 3c; chr13:18,645,918–18,653,936), SETBP1 (SET Binding Protein 1, chr18:40,535,138–40,898,771), ZNF618 (Zinc Finger Protein 618; chr9:115,678,383–115,858,696), MDGA1 (MAM Domain Containing Glycosylphosphatidyl Inositol (GPI) Anchor 1; chr6:37,708,262–37,773,744), MESP2 (Mesoderm Posterior Basic Helix-Loop-Helix Transcription Factor 2; chr15:88,120,593–88,122,986), FHAD1 (Forkhead-Associated (FHA) Phosphopeptide Binding Domain 1; chr1:15,446,355–15,597,209), and COL24A1 (Collagen, Type XXIV, Alpha 1; chr1:85,967,504–86,394,742) (Table 2). In this collection of susceptibility genes, NEBL (rs16921209 and rs7922552) and MDGA1 (rs12210919 and rs12211370) both have been hit only by two significant genetic polymorphisms from the top ranking list of mutations, whereas the genetic variant, rs17076896, is shared in the upstream promoter region between TUBA3C and LOC101928697. The putative functional roles of these genes in the formation of CAAs in KD will be discussed afterward.

**Table 2:** Top 15 non-intergenic SNPs associated with susceptibility of CAA formation in KD.

SNP	LOCUS	Allele (D/d)	F_A	F_U	P	OR	CR	HWE	TYPE	SYMBOL
rs16921209	10:21208109	C/G	0.182	0.007	7.44E-09	32.22	100.00%	0.0475	intron	NEBL
rs17076896	13:18654063	A/G	0.227	0.014	8.04E-09	21.03	99.36%	1.0000	promoter	TUBA3C
rs7922552	10:21225030	G/C	0.182	0.007	8.43E-09	32.00	99.36%	0.0478	intron	NEBL
rs17782904	18:40567708	C/T	0.136	0.003	8.24E-08	45.95	100.00%	0.0191	intron	SETBP1
rs11793049	9:115813690	T/A	0.091	0.000	2.36E-07		100.00%	1.0000	intron	ZNF618
rs12210919	6:37753490	T/C	0.227	0.021	3.67E-07	14.02	100.00%	1.0000	intron	MDGA1
rs12900413	15:88122043	T/C	0.364	0.062	7.18E-07	8.70	100.00%	1.0000	intron	MESP2
rs10127456	1:15526459	C/T	0.182	0.014	1.39E-06	16.00	100.00%	0.0873	intron	FHAD1
rs12211370	6:37757322	C/T	0.227	0.024	1.62E-06	11.97	100.00%	1.0000	intron	MDGA1
rs1842579	1:86061670	G/A	0.318	0.051	2.27E-06	8.62	100.00%	0.5464	intron	COL24A1

The major SNP allele frequency in cases and controls were indicated in the F_A and F_U columns, respectively. OR and CR represent the odds ratio and call rate. The p-values of Hardy-Weinberg equilibrium were indicated in the HWE column.

In addition to association analyses based upon individual major and minor alleles, the distribution of allele types (DD, Dd, and dd indicating major homozygous, heterozygous, and minor homozygous, respectively) were put into dominant, recessive, and general genotypic model analyses to identify the difference between cases and controls. Hence, the hit list of top ranking SNPs ordered by p-values computed using genotypic model has been identified and involved in genes including KCNH7 (Potassium Channel, Voltage Gated Eag Related Subfamily H, Member 7), TRAF5 (TNF Receptor-Associated Factor 5), NDUFA5 (NADH Dehydrogenase (Ubiquinone) 1 Alpha Subcomplex, 5), and MDGA2 (MAM Domain Containing Glycosylphosphatidyl-inositol (GPI) Anchor 2) (Table 3). When considering inheritance patterns, rs981840 (*P* = 2.93 × 10^−5^) and rs4383352 (*P* = 3.06 × 10^−5^) in KCNH7 and rs10137971, rs34362363, rs4900777, rs6572432, and rs7155197 (all p-values of 5.36 × 10^−5^) in MDGA2 were qualified as significant in dominant inheritance model, whereas rs4951523 (*P* = 3.34 × 10^−5^) and rs7550702 (*P* = 3.34 × 10^−5^) in TRAF5 and rs12666974 (*P* = 3.34 × 10^−5^) in NDUFA5 were more significant in recessive inheritance model.

**Table 3:** Top 10 non-intergenic SNPs associated with susceptibility of CAA formation in KD using the general genotypic model.

SNP	LOCUS	TYPE	SYMBOL	G_A	G_U	G_P	LABEL
rs981840	2:163129567	intron	KCNH7	4/7/0	7/61/78	2.93E-05	D
rs4383352	2:163199542	intron	KCNH7	4/7/0	7/62/77	3.06E-05	D
rs4951523	1:209596667	intron	TRAF5	8/2/1	19/68/59	3.34E-05	R
rs7550702	1:209596192	intron	TRAF5	8/2/1	19/68/59	3.34E-05	R
rs12666974	7:122979210	intron	NDUFA5	5/6/0	12/62/72	5.15E-05	R
rs10137971	14:47045511	intron	MDGA2	0/0/11	19/77/50	5.36E-05	D
rs34362363	14:47016756	intron	MDGA2	0/0/11	19/77/50	5.36E-05	D
rs4900777	14:47044337	intron	MDGA2	0/0/11	19/77/50	5.36E-05	D
rs6572432	14:47036604	intron	MDGA2	0/0/11	19/77/50	5.36E-05	D
rs7155197	14:47045336	intron	MDGA2	0/0/11	19/77/50	5.36E-05	D

The patients with different genotypes (ordered as dd/Dd/DD representing minor homozygous, heterozygous, and major homozygous, respectively) in cases and controls were represented as G_A and G_U, respectively. The p-values of genotypic tests using Fisher’s exact test in 2 degree of freedom were shown as G_P. Corresponding inheritance patterns have been indicated as D (dominant) or R (recessive) in the LABEL column.

### NEBL genetic polymorphisms may be related to KD-associated CAA complications

**Fig 2:**
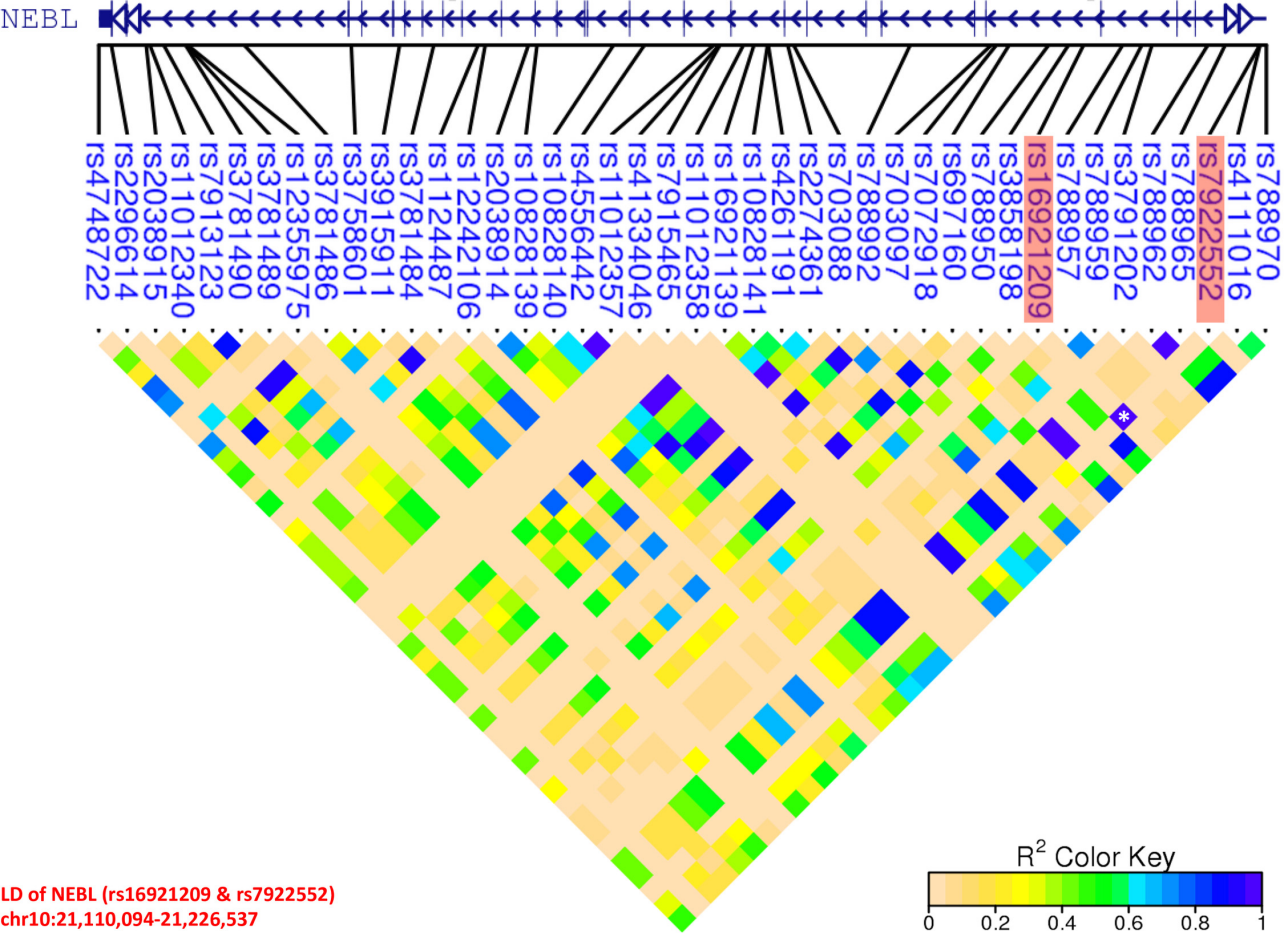
Linkage disequilibrium plot of a region covering all SNPs of NEBL. Two significant SNPs; rs16921209 (P = 7.44 × 10−9; OR = 32.22) and rs7922552 (P = 8.43 × 10−9; OR = 32.0), both located in the introns of NEBL are in high linkage disequilibrium (r^2^ = 0.99), which might imply their co-interplay to susceptibility of CAA formation in KD patients.

Nebulette protein has been reported to encode a 109 kDa nebulin homologous protein that is considerably expressed in cardiac muscle and is specifically localized in the sarcomeric Z-line of the heart [50]. Genetic variants in NEBL have been indicated to be causative for the occurrence of sudden cardiac death (SCD), in which an abrupt loss of heart function results in a sudden, unexpected death [51]. Moreover, it has been demonstrated to involve in mechanosensing and facilitate generation via the association with actin and tropomyosin-troponin complex [52]. In vitro and vivo studies, both have suggested its critical role in the stabilization of the thin-filament-Z-line alignment [50]. Missense abnormalities in NEBL have been illustrated to attribute to dilated cardiomyopathy (DCM) and endocardial fibroelastosis in humans and animal models [53]. Additionally, the observation of up-regulating genes responsive to cardiac stresses on nebulette-deficient mice has indicated its dispensable role on the regulation of normal cardiac function [50]. In this study, two genetic variants: rs16921209 (*P* = 7.44 × 10^−9^; OR = 32.22) and rs7922552 (*P* = 8.43 × 10^−9^; OR = 32.0) in the intronic regions of NEBL were revealed as significant SNPs to susceptibility of CAA formation in KD patients (Table 2). Furthermore, in linkage disequilibrium (LD) analysis, they were found to be in strong LD (r^2^ = 0.99; Fig 2) at 10p12.31, in which the enclosed genomic region comprises multiple qualified SNP hits in the upstream introns of NEBL. As a result, this might imply that NEBL also play an important role on the development of CAAs during KD progression.

### TUBA3C genetic variants to susceptibility of CAA occurrence in KD patients

**Fig 3:**
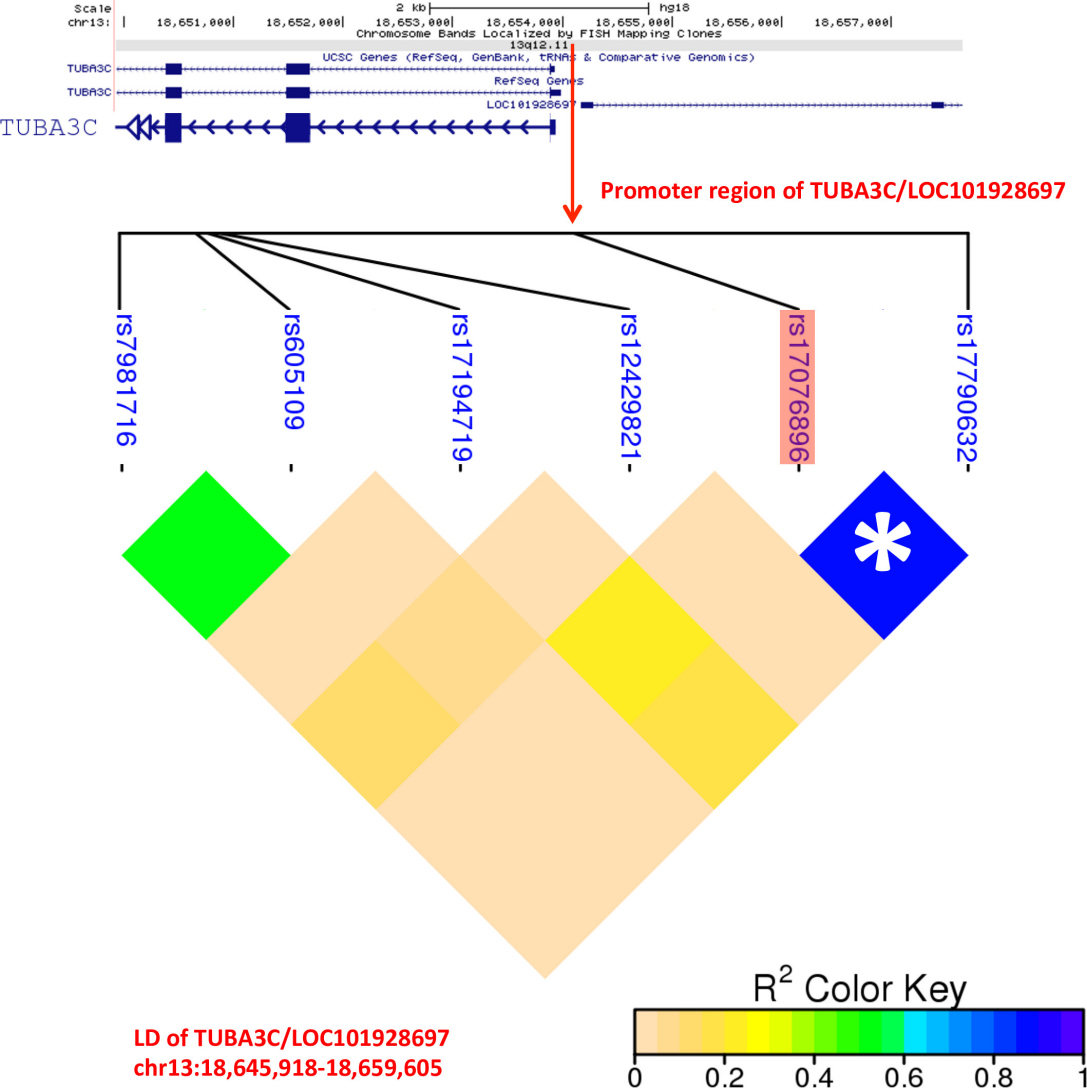
Linkage disequilibrium plot of a region containing all SNPs of TUBA3C and LOC101928697. The significant genetic variant of rs17076896 (*P* = 8.04 × 10−9; OR = 21.03) is located in the shared promoter region between TUBA3C and LOC101928697, which showed a high linkage disequilibrium (r^2^ = 0.90) with another down-stream SNP, rs17790632, in LOC101928697.

The susceptibility variant of rs3818298 in T-Complex 1 (TCP1) has first been reported by *Burgner et al*. [14] to associate with KD risk and recently *Lou et al*. [31] has confirmed the pattern with additional independent cohort in a Chinese population. The protein encoded by TCP1 plays an important role in a member of the chaperonin of TCP1 ring complex in the functions of interacting with and structurally folding actin and tubulin. Instead of identification of TCP1, another tubulin family gene, TUBA3C has been identified with a significant SNP of rs17076896 (*P* = 8.04 × 10^−9^; OR = 21.03) located on its upstream promoter region in KD group with CAA formation (Table 2). To share the same promoter region, another uncharacterized gene, LOC101928697, on the downstream region with divergent transcription direction to TUBA3C might imply its putative function associated with the risk of CAA in KD patients. Additionally, the rs17790632 SNP in LOC101928697 has shown a high linkage pattern with rs17076896 in the shared promoter region with TUBA3C (r^2^ = 0.90; Fig 3) in LD analysis, which it reflects that LOC101928697 might also serve as susceptibility gene to CAA formation during KD progression.

### TNF- α and MMP associated genes may play a role in the formation of CAAs in KD

It has been reported that tumor necrosis factor α (TNF-α) plays a crucial role of inflammatory response in KD patients via up-regulating the transcription of matrix metalloproteinases (MMP) such as MMP-9, resulting in increased vessel wall elastin degradation and CAA formation [12]. In this study, we did reveal several associated, co-functional genes to susceptibility of this regulatory pathway. On one hand, two out of three qualified genetic variants (rs4951523 (*P* = 3.34 × 10^−5^) and rs7550702 (*P* = 3.34 × 10^−5^)) in TRAF5 (TNF Receptor-Associated Factor 5) have been associated with inheritance patterns as recessive genotype models (Table 3). It has been shown that TRAF5 is an important, versatile mediator beyond the TNFR-superfamily (SF); including viral mimics of its members, mediating specific cytokine receptor signals and innate immune receptor, as well as signal transductions of the T-cell receptor (TCR) complex [54]. Therefore, this reflects again that TNF and its co-regulatory genes like TRAF5 might play a crucial role in coronary artery disease.

Alternatively, it has been demonstrated by both animal models and clinical studies, matrix metalloproteinases (MMPs) such as MMP-9 are putative biomarkers for the function of cardiac remodeling, which are regulated by inflammatory signals to mediate changes in extracellular matrix [12]. Furthermore, within this family, some membrane-type MMPs (MT-MMPs) with lacks of an additional transmembrane domain or a small cytoplasmic tail, need to deliver signals by attaching to the plasma membrane using glycosylphosphatidyl-inositol (GPI) anchor proteins. In allelic association analysis, two specific SNPs of rs12210919 (*P* = 3.67 × 10^−7^; OR = 14.02) and rs12211370 (*P* = 1.62 × 10^−6^; OR = 16.00) in MDGA1 (MAM Domain Containing GPI Anchor 1) were identified as significant genetic variants in our top-ranking list (Table 2). Meanwhile, in general genotypic association analysis, at least five SNPs of rs10137971, rs34362363, rs4900777, rs6572432, and rs7155197 (all p-values of 5.36 × 10^−5^) in MDGA2 were defined as significantly dominant inheritance patterns (Table 3). Taken together, it might imply that MDGA gene family functions a putative regulatory role in association with the formation of CAA in KD patients.

### GO cluster enrichment analysis revealed consistent results as in genetic association analysis

**Fig 4:**
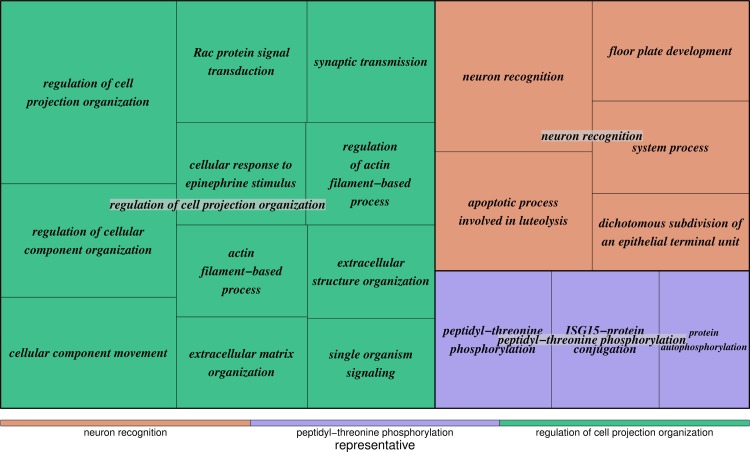
Top three gene ontology enrichment groups associated with susceptibility of CAA formation in KD. After gene ontology (GO) enrichment analysis, 162 over-represented GO terms were obtained and further summarized into GO clusters. The top three significant clusters were regulation of cell projection organization (*P* = 2.22 × 10^−6^), neuron recognition (*P* = 6.72 × 10^−5^), and peptidyl-threonine phosphorylation (*P* = 1.53 × 10^−3^).

To focus on investigating the underlying mechanism of developing CAAs in KD patients, 263 protein-coding genes hit by 720 significant SNPs qualified from our genetic association analysis were collected, and gene ontology (GO) enrichment analysis has been applied to those susceptibility genes to determine over-represented GO terms. Accordingly, in total 162 enriched GOs were acquired and further summarized into higher levels of representative GO clusters to facilitate the interpretation of major functions related to CAA formation during KD progression upon genetic variants. Among the susceptibility genes highlighted above, NEBL and TUBA3C were associated with the GO clusters of regulation of cellular component size and protein complex assembly, respectively. TRAF5 was related to apoptotic process/death/positive regulation of cell proliferation and MDGA1/2 were contributed to neuron differentiation. The top three representative GO clusters were regulation of cell projection organization (*P* = 2.22 × 10^−6^), neuron recognition (*P* = 6.72 × 10^−5^), and peptidyl-threonine phosphorylation (*P* = 1.53 × 10^−3^) (Fig 4). For regulation of cell projection organization, most GO terms in this cluster were composed of cellular and extracellular organizations, involving in actin related genes. As a result, the global pattern has shown that genes involved in the formation of muscle cells like actin might function importantly in the development of CAAs in KD patients.

Potassium (K^+^) channels are the most heterogeneous and extensively recognized class of ion channels, and are widely identified as putative therapeutic targets in the treatment of neuron diseases like multiple sclerosis. They have been reported to be involved in a variety of cell types underlying both normal and pathophysiological processes, including nerve impulse propagation, muscle contraction and cellular activation [55]. They are comprised of dynamic pore-forming transmembrane proteins that selectively allow the flow current of potassium ions down an electrochemical gradient. In KD community, susceptibility genetic variants contained in Potassium channels associated genes have been reported to show a connection with the complication of CALs in KD patients [35]. In this study, a quantity of Potassium channels associated genes has been identified by containing significant SNPs to susceptibility of the development of CAAs in KD patients, resulting in the identification of neuron recognition in GO enrichment analysis (Fig 4). Hence, this represents that Potassium channels associated genes might facilitate CAA formation during KD progression.

For the over-representative cluster of peptidyl-threonine phosphorylation (Fig 4), one member gene TGFBR2 (Transforming Growth Factor, Beta Receptor II) has been reported to associate with the pathogenesis of KD and CALs [56]. Meanwhile, another member gene CAMK2D (Calcium/Calmodulin-Dependent Protein Kinase II Delta) has been demonstrated to involve in a plausible biological network and to decrease transcript abundance in the acute phase of KD [14, 31]. Therefore, some critical genes identified to act significantly in KD with CALs might also perform alternative role in the pathogenesis of CAAs in KD patients.

## Discussion and Conclusion

Due to the obscurity of the causes resulting in KD, there is no existing clinical method to prevent its occurrence. Moreover, KD remains the leading cause of acquired heart damages in children younger than 5 years old. Many studies have been dedicated to investigate critical factors in the development of CAAs during KD progression. Briefly, in 2005 Matsubara *et al*. reported that histological findings in KD including vasculitis, endothelial necrosis, and infiltration of mononuclear cells into blood vessels [57]. In previous studies, plasma levels of inflammatory cytokines (TNF-α, IL-4, IL-5, IL-6, IL-17, IL-31, and IP-10) [7, 8, 58–62], chemokines and adhesion molecules were elevated at the acute stage of KD [57]. Macrophage colony-stimulating factor (M-CSF) has been performed to play a critical role in the pathogenesis of KD and can be used as an indicator for the risks of valvulitis and coronary arteritis [11, 63]. Guiducci *et al*. reported that microparticles (MPs) may develop from endothelial damage and cell activation is significantly increased as well as endothelial cells and T cells are the major sources [64]. Taken together, macrophages and platelets also get recruited to this site of vasculitis or may play a role in the immunopathogenesis of KD.

In this study, a comprehensive GWAS has been conducted upon 157 valid KD patients to identify susceptibility genetic polymorphisms and their corresponding genes as major risk factors to CAA formation in KD patients. Although potential links between susceptibility loci identified in this study and the formation of CAAs in KD patients still remain gaps for researchers to clarify. However, this study provides various hints for researchers in KD community to postulate that the essential underlying mechanism of the pathogenesis of CAAs in KD might be associated with genes related to cardiac muscles and vessels. Accordingly, our results indicate that susceptibility genes (e.g., NEBL, TUBA3C, TRAF5, and MDGA1/2) carried with certain significant genetic polymorphisms might play important roles in the risk of CAA complications in KD patients from genetic perspective. Furthermore, from GO perspective, these susceptibility loci were enriched in the functions related to regulation of cell projection organization, neuron recognition, and peptidyl-threonine phosphorylation.

To examine the connection between genetic alternations and gene expression, the susceptibility genes highlighted in this study were investigated in recent transcriptome studies related to KD CAA. For instance, according to the 1,074 DEGs reported by Rowley *et al*. [65], most of our reported genes with significant genetic variants were not classified as dysregulation ones. Accordingly, the authors claimed that most genes in cytokine and growth factor family might not play the most prominent role in KD CAA from the transcriptome perspective. However, as explained in the same study, the interplay between genetic alternations, gene expression, and protein production are not always associated with each other. Therefore, more integrative studies (genomics, transcriptomics and proteomics together) will be needed to help us understand the underlying pathogenesis of KD CAA.

Recently, IVIG treatment has been demonstrated to associate with the inhibition of TNF-α-induced MMP9 expression and shows a protective effect in KD CAA [66]. Some IVIG-resistant patients have been reported with a higher risk of developing coronary artery abnormalities in KD [67]. Infliximab, as an anti-cytokine therapy, leverages the blocking of TNF-α pathway to provide another treatment solution to KD patients with IVIG resistance [68]. Therefore, although in the recent transcriptomic study, genes from TNF-α family were shown as no up-regulation in KD CAA [65], genetic alternations [69], animal models [59, 70–72], and clinical therapy [66, 68] associated with these genes have been keeping to report as susceptibility factors and consequences to KD CAA. This remains an open question about the interplay among different levels to the pathogenesis of KD CAA. As a result, our results help to gain insights into the potential routes of the development of CAAs in KD and might benefit researchers in KD community to improve the diagnosis and prognosis of KD in personalized medicine.

## Supporting Information

S1 File GWAS table containing all non-intergenic SNPs of KD CAA in this study. Major (D) and minor (d) alleles were shown in the Alleles column. The major SNP allele frequency in cases and controls were indicated in the F_A and F_U columns, respectively. OR represents the odds ratio. The p-values of Chi-squared test were indicated in the P column.
(XLSX)
